# Impact of interval between induction of spinal anesthesia to delivery on umbilical arterial cord ph of neonates delivered by elective cesarean section

**DOI:** 10.1186/s12884-022-04536-y

**Published:** 2022-03-17

**Authors:** Alaa S. Hassanin, Hazem F. El-Shahawy, Sherif Hanafi Hussain, Ahmed M. Bahaa Eldin, Marwa Mohammed Elhawary, Mohamed Elbakery, Mohammed S. E. Elsafty

**Affiliations:** 1 Department of Obstetrics and Gynecology, Ain Shams Maternity Hospital, Faculty of Medicine, Ain Shams University, 38 Ramsis Street, Abbasiya, 11591 Cairo, Egypt; 2 Department of Anesthesia and Intensive Care, Faculty of Medicine, Azhar University, Cairo, Egypt

**Keywords:** Neuraxial anesthesia, Umbilical cord arterial pH, Cesarean delivery, Fetal distress, Spinal anesthesia, Time interval

## Abstract

**Background:**

To evaluate the impact of interval between induction of spinal anesthesia to delivery of the fetus by elective cesarean section on umbilical arterial pH and neonatal outcome.

**Patients and methods:**

Two hundred and twenty pregnant women who were planned for elective cesarean section at term under spinal anesthesia were recruited. Minimum systolic, diastolic and mean arterial blood pressures (SBP, DBP, MAP) and largest pressure decrease (SBP, DBP, MPA) were also recorded. Induction of spinal anesthesia to delivery interval was measured. Following delivery, umbilical arterial cord analysis for pH and base deficit were done. Apgar scores at 1 min and at 5 min, neonatal intensive care unit (NICU) admission, need for mechanical ventilation and incidence of hypoxemic-ischemic encephalopathy were recorded.

**Results:**

Induction of spinal anesthesia to delivery interval was 25.7 ± 5.6 min. Lowest SBP and MAP reached during cesarean delivery were 88.9 ± 7.3 mmHg and 60.4 ± 5.6 mmHg, respectively. MAP < 65 mmHg was reached in 136 (62%) patients with a decrease of MAP of > 20% in 149 (68%) patients. Duration of the longest hypotension episode was 3.3 ± 2.2 min. All patients required ephedrine administration for hypotensive episodes with an average dosage of 11.4 ± 3.2 mg. Umbilical pH of 7.3 ± 0.1 and base deficit of 8.3 ± 4.4 mmol/l were recorded. Apgar scores at 5 min were 8.5 ± 1.2. Eight (3.6%) neonates were admitted in the NICU. One neonate needed mechanical ventilation. There were no cases of hypoxemic-ischemic encephalopathy. There were inverse correlations between induction of spinal anesthesia to delivery interval, body mass index (BMI) and duration of longest hypotension episode in relation to umbilical pH (*r* = -0.817, -0.395 and -0.268, respectively). Cut off value for induction of spinal anesthesia to delivery interval greater than 27 min predicted an umbilical pH of < 7.2. Cut off value for the duration of the longest hypotension episode greater than 5 min predicted an umbilical pH of < 7.2. Cut off value for BMI greater than 35 kg/m^2^ predicted an umbilical pH of < 7.2.

**Conclusion:**

Prolonged interval between induction of spinal anesthesia to delivery could be associated with neonatal acidosis. This could be aggravated by maternal obesity and prolonged duration of hypotension episodes during cesarean delivery.

## Introduction

The selection of appropriate anesthesia for cesarean section depends on various factors such as indication for cesarean delivery, emergency status, maternal status, and patient preference. The effect of anesthesia on uterine blood flow and uterine vascular resistance, hence affecting placental perfusion pressure, can directly impact the outcome of pregnancy [1].

Because of the rapid onset of adequate anesthesia, the avoidance of fetal exposure to anesthetic drugs, and the maternal risks associated with general anesthesia, spinal anesthesia is the most commonly used technique for planned cesarean delivery [2]. Among the most serious risks of spinal anesthesia is maternal hypotension during cesarean delivery. When prophylactic vasopressor infusions are not used, spinal hypotension occurs in up to 74 percent of planned cesarean deliveries and is linked to fetal acidosis [2].

There is a link between the drop of maternal blood pressure (BP) and fetal acidosis [3]. Yet, the impact of the duration of hypotension episodes is not well investigated. Fetal distress and adverse neonatal outcomes are usually not expected during an elective cesarean section when compared to emergency cesarean section performed during labor. The time from the administration of spinal anesthesia to the extraction of the fetus is the window of potential fetal compromise [3].

During this pre-delivery window, both maternal hypotension and prolonged intraoperative time intervals contribute to a poor neonatal outcome, but their relative clinical importance and critical thresholds require further study [4].

Comparison of neonatal outcomes who were exposed to various anesthesia methods is not well studied. The relatively common criteria for evaluating the infant's clinical status after delivery is the first and fifth-minute Apgar scoring system [3]. Umbilical cord arterial blood assessment is considered the gold standard investigation for determining the acid–base status of the neonate and therefore determining the performance of the placenta. Furthermore, the Be, Hco3, Pco2, and pH values in the umbilical artery blood show the fetal state at delivery, especially in high-risk pregnancies, as well as the risk of fetal distress [2].

Maternal obesity can aggravate neonatal and umbilical arterial pH depression caused by neuraxial anesthesia-related hypotension due to reduction of uterine perfusion during cesarean Sect.  [5]. Intraoperative hypotension alone has been reported to predict lower neonatal umbilical arterial pH independently after removing maternal obesity as a confounding factor. These factors may compound one another because obese women undergoing cesarean delivery have been shown to have a greater incidence of intraoperative hypotension [6].

This study aimed to evaluate the impact of interval between induction of spinal anesthesia to delivery of the fetus by elective cesarean section on umbilical arterial pH and neonatal outcome including 5 min Apgar score and need for NICU admission for respiratory depression.

## Patients and methods

This was a prospective observational study conducted between 1^st^ of December, 2019 to 30^th^ of November, 2020 in Ain Shams Maternity Hospital, Cairo, Egypt. Two hundred and twenty pregnant women who were planned for elective cesarean delivery at term under spinal anesthesia were recruited. All patients had an ultrasound and a reassuring non-stress cardiotocograph before delivery to exclude patients with pre-existing fetal distress. All patients who were pregnant with multiple fetuses or fetuses with congenital anomalies or women who went into labor and had emergency cesarean deliveries were excluded; so were patients who had medical or obstetrical complications with pregnancy and patients who were planned for additional surgeries with cesarean section. Approval from Ain Shams Maternity Hospital Ethical Committee was obtained and all participants signed written informed consents for participation in the study.

All participants were monitored primarily before induction of spinal anesthesia and base-line systolic blood pressure (SBP), diastolic blood pressure (DBP), mean arterial pressure (MAP), base-line heart rate and base-line oxygen saturation were recorded. All patients received a preload of 1000 ml of Ringer lactate through an 18 gauge cannula. Standard approach for spinal anesthesia was performed in a flexed, sitting position using a 25-gauge Sprotte needle placed in the L2–L3 or L3–L4 intervertebral space through which 10 mg of hyperbaric 0.5% bupivacaine and 25mcg of fentanyl were injected. The dose was reduced to 1.75 mL of hyperbaric 0.5% bupivacaine in the patients with a height less than 1.55 m. After induction of spinal anesthesia women were placed in the left lateral tilt position (10-15degrees) until pfannensteil incision was performed and the blood pressure was monitored every 3–5 min from the beginning of anesthesia until fetal delivery and any episode of hypotension (defined by SBP less than 100 mmHg or MAP less than 65 mmHg) was recorded. Episodes of hypotension were managed by infusion of 500 cc of Ringer lactate and fixed incremental doses of ephedrine by 3–6 mg each time according to the severity of hypotension, which was also recorded. Minimum blood pressure (SBP, DBP, MAP) and largest pressure decrease (SBP, DBP, MPA) were also recorded. Induction of spinal anesthesia to delivery interval was measured from injection of bupivacaine through the spinal needle until delivery of the fetus outside the uterus. Induction of spinal anesthesia to skin incision interval was measured from injection of bupivacaine in the spinal needle until start of the pfannensteil skin incision. Skin incision to uterine incision interval was measured from start of pfannensteil skin incision until start of uterine incision. Uterine incision to delivery interval was measured from start of uterine incision until delivery of fetus outside the uterus.

Umbilical artery samples were obtained after delivery using pre-heparinized 3 ml syringe from fetal umbilical artery. To avoid inclusion of inadvertent umbilical venous gases samples, samples in which oxygen pressure was > 60 mm Hg were excluded. Following delivery, fetal birth weight (grams), umbilical cord arterial analysis for pH, pCO2 and base deficit were done. Apgar scores at 1 min and at 5 min were evaluated by the pediatrician. The primary study outcome was neonatal acidosis defined as pH less than 7.2 or base deficit of more than 12.0. The secondary outcome was a composite of 5-min Apgar score of < 7, transient tachypnea of the newborn, respiratory distress syndrome, hypoxemic-ischemic encephalopathy and admission to the neonatal intensive care unit.

## Statistical analysis

IBM-SPSS Statistics for windows, version 23.0 (Copyright IBM Corp., Armonk, N.Y., USA. 2015) was used data analysis. Descriptive statistics including mean, standard deviation, median, range and percentage were calculated. Chi-square test was utilized to compare the discrepancy in distribution of frequencies among various groups. Analysis of variance [ANOVA] tests were used for comparison among different times in the same group in quantitative data. Linear Correlation Coefficient [r] was utilized for identification of a correlation between two quantitative variables in a single group. Independent t-test was used to distinguish the means of dichotomous data for continuous variables. Receiver Operating Characteristic (ROC) curve was utilized to select the best cut-off point for the test. Kruskal–Wallis Test, a nonparametric equivalent to one-way ANOVA, evaluated whether several independent samples were from the same population. If an underlying variable had a continuous distribution, and required an ordinal level of measurement, Logistic Regression Binary Logistic Regression models were performed. A *p*-value ≤ 0.05 was evaluated as significant.

## Results

220 patients were recruited. They were 29.4 ± 4.1 years of age with an average BMI of 33.5 ± 2.8 kg/m^2^. They all had elective lower segment cesarean sections between 38 and 39 weeks for different indications, of which previous lower segment cesarean section was the most frequent (79%). Induction of spinal anesthesia to delivery interval was 25.7 ± 5.6 min, while skin incision to uterine incision interval was 7.1 ± 2.4 min. Uterine incision to delivery interval was 1.2 ± 0.5 min with 87% of patients delivered in cephalic presentation. All patients required ephedrine administration for hypotensive episodes with an average dosage of 11.4 ± 3.2 mg. New born babies had an average weight of 3335.7 ± 206.7gm and an umbilical PH of 7.3 ± 0.1 with base deficit of 8.3 ± 4.4 mmol/l. Mean Apgar scores at 5 min were 8.5 ± 1.2. Eight (3.6%) neonates were admitted in the NICU due to respiratory distress and there were no cases of hypoxemic-ischemic encephalopathy (Table 1). Five neonates were diagnosed as transient tachypnea of the newborn and resolved with nasal oxygen. The other two neonates were treated with continuous positive airway pressure (CPAP). Umbilical PH ranged between < 7 and 7.2. One neonate suffered from severe respiratory distress syndrome and required mechanical ventilation. Umbilical PH for this neonate was < 7. All neonates improved and were discharged. 164 (75%) patients had an induction of spinal anesthesia to delivery interval of 20 to 29 min, while 180 (82%) patients delivered neonates with pH equal to or > 7.21. Baseline SBP and MAP before start of spinal anesthesia was 110.9 ± 8.0 mmHg and 82.2 ± 5.3 mmHg, respectively, and lowest SBP and MAP reached during cesarean delivery was 88.9 ± 7.3 mmHg and 60.4 ± 5.6 mmHg, respectively. MAP < 65 mmHg was reached in 136 (62%) patients with a decrease of MAP of > 20% in 149 (68%) patients. Duration of the longest hypotension episode was 3.3 ± 2.2 min (Table 2).

**Table 1:** Descriptive Data of Participants

Maternal data:	Range	Mean ± SD
Age (years)	21–41	29.84 ± 4.11
BMI (kg/m^2^)	24–39	33.45 ± 2.75
Parity	0–4	2
Indication of CS:	**N**	**%**
RLSCS	173	(78.6%)
Breech	29	(13.2%)
Maternal request	15	(6.8%)
Previous Intrauterine fetal demise	3	(1.4%)
Previous cerebral palsy	0–4	1
Intraoperative data:	**Range**	**Mean ± SD**
Induction of spinal anesthesia to delivery interval (min)	14–50	25.69 ± 5.56
Induction of spinal anesthesia to skin incision interval (min)	9–28	17.34 ± 3.70
Skin incision to uterine incision interval (min)	3–20	7.06 ± 2.35
Uterine incision to delivery interval (min)	1–5	1.18 ± 0.53
Cephalic presentation	**N-%:** 191 (86.8%)	
Need for vasopressor (ephedrine)	**N-%:** 220 (100.0%)	
Dose of vasopressor (ephedrine) (mg)	4–21	11.43 ± 3.20
New born data:		
Umbilical PH	7–7.36	7.29 ± 0.08
Base deficit (mmol/l)	0.3–21.8	8.34 ± 4.43
Base deficit < 12 mmol/l	**N-%:** 183 (83.2%)	
Fetal birth weight (gm)	2500–4200	3335.68 ± 206.65
Apgar 1 min	5–9	8.18 ± 0.81
Apgar 5 min	5–10	8.52 ± 1.18
Apgar 5 min < 7	**N-%:** 4 (1.8%)	
NICU admission	**N-%:** 8 (3.6%)	
Need for intubation/ventilation	**N-%:** 1 (0.5%)	
Hypoxic ischemic encephalopathy	0

**Table 2:** Descriptive Data of Participants

Induction of spinal anesthesia to delivery (min)	N	%
< 20	18	8.2
20–29	164	74.5
30–39	33	15.0
40 or more	5	2.3
**Umbilical PH**	**N**	**%**
< 7.0	4	1.8
7.01–7.10	6	2.7
7.11–7.2	30	13.6
7.21–7.3	57	25.9
> 7.3	123	55.9
**Maternal blood pressure data:**	**Range**	**Mean ± SD**
**Baseline SBP (mmHg) ( just before induction of spinal anesthesia)**	90–125	110.87 ± 8.02
**Minimum SBP (mmHg)**	70–120	88.86 ± 7.33
**Decrease in SBP (mmHg)**	0–55	22.01 ± 9.75
**Decrease SBP > 20%**	**N-%:** 101 (45.9%)	
**Decrease SBP > 40%**	**N-%:** 6 (2.7%)	
**Baseline MAP (mmHg) ( just before induction of spinal anesthesia)**	70–93	82.16 ± 5.27
**Minimum MAP (mmHg)**	44–75	60.35 ± 5.61
**Decrease in MAP (mmHg)**	5–40	21.70 ± 7.08
**MAP < 65 mmHg**	**N-%:** 136 (61.8%)	
**Decrease MAP > 20%**	**N-%:** 149 (67.7%)	
**Decrease MAP > 40%**	**N-%:** 7 (3.2%)	
**Duration of the longest hypotension episode (min)**	0–9	3.35 ± 2.19

As shown in Table 3, umbilical pH less than 7 was associated with induction of spinal anesthesia to delivery interval of 40.5 ± 4.4 min, induction of spinal anesthesia to skin incision interval of 24.3 ± 2.9 min, skin incision to uterine incision interval of 13.8 ± 6.2 min and uterine incision to delivery interval 2.3 ± 0.5 min. Comparing these intervals at an umbilical pH of > 7.3, they were 22.5 ± 3.4 min, 15.3 ± 2.7 min, 6.2 ± 1.4 min and 1.1 ± 0.2 min, respectively, which showed a highly statistically significant difference (*P* < 0.001). Other data related to different levels of umbilical pH were BMI, drop in SBP and MAP, duration of longest hypotension episode, degree of base deficit and Apgar scores in neonates (Table 4). BMI of 35.5 ± 3.4 kg/m^2^ was associated with umbilical artery pH of 7.01–7.10 compared to lower BMI of 34 ± 2.0 kg/m^2^ in women delivering neonates with umbilical pH of 7.21–7.3, which showed highly statistically significant difference (*p* < 0.001). Similarly, a drop of SBP and MAP of 30 ± 21.9 mmHg and 28.9 ± 5.7 mmHg, respectively, were associated with an umbilical artery pH of 7.01–7.10, compared to drop of SBP and MAP of 23.7 ± 9.0 mmHg and 24.8 ± 6.3 mmHg, respectively, were associated with umbilical pH of 7.21- 7.3, which showed statistically significant difference (*p* = 0.017 and *p* < 0.001, respectively). At an umbilical pH of 7.01–7.10, duration of longest hypotension episode was 4.3 ± 2.4 min compared to 3.5 ± 1.1 min at an umbilical pH of 7.21–7.3 (*p* = 0.012). Apgar scores at 1 min were 7.8 ± 1 at umbilical pH of 7.01–7.10 compared to 8.2 ± 0.8 at umbilical pH of 7.21–7.3 (*p* = 0.002) (Table 4).

**Table 3:** Relation between Umbilical pH and Different Studied Intervals

	**Umbilical pH**						
	** < 7.0**	**7.01–7.10**	**7.11–7.2**	**7.21–7.3**	** > 7.3**	ANOVA	
						f	*P*-value
**Induction of spinal anesthesia to delivery interval (min)**	40.50 ± 4.36	35.17 ± 4.92	32.17 ± 5.55	27.07 ± 2.27	22.53 ± 3.38	78.418	< 0.001**
**Induction of spinal anesthesia to skin incision interval (min)**	24.25 ± 2.87	22.67 ± 3.08	21.10 ± 3.13	18.81 ± 2.33	15.26 ± 2.72	49.450	< 0.001**
**Skin incision to uterine incision interval (min)**	13.75 ± 6.24	10.17 ± 3.31	8.97 ± 2.72	7.05 ± 1.71	6.24 ± 1.37	29.618	< 0.001**
**Uterine incision to delivery interval (min)**	2.25 ± 0.50	2.17 ± 0.98	1.57 ± 0.94	1.14 ± 0.44	1.06 ± 0.23	13.650	< 0.001**

**Table 4:** Relationship between Various Parameters and Umbilical pH

	**Umbilical pH**						
	** < 7.0**	**7.01–7.10**	**7.11–7.2**	**7.21–7.3**	** > 7.3**	**Tests**	
						**f/X**^**2**^	***P***-value
**Age (years)**	28.75 ± 5.38	29.50 ± 5.21	31.20 ± 3.75	30.12 ± 3.66	29.43 ± 4.29	1.278	0.279
**BMI (kg/m2)**	33.75 ± 3.86	35.50 ± 3.39	35.57 ± 3.35	33.95 ± 2.04	32.59 ± 2.45	10.285	< 0.001*
**Parity**	1.5(1–3)	1.5(1–4)	2(0–3)	2(0–3)	1(0–4)	4.606	0.330
**Frequency of previous abortions**	0.5(0–1)	0(0–2)	0(0–1)	0(0–2)	0(0–3)	1.731	0.785
**Previous CS**	1.5(1–3)	1.5(1–3)	2(0–3)	2(0–3)	1(0–4)	4.134	0.388
**Baseline SBP (mmHg) ( just before** **induction of spinal anesthesia)**	107.5 ± 5	114.50 ± 8.46	113.00 ± 7.61	110.37 ± 7.49	110.51 ± 8.36	1.134	0.341
**Minimum SBP (mmHg)**	87.5 ± 13.23	84.5 ± 15.73	88.43 ± 12.56	86.67 ± 6.65	90.24 ± 4.35	3.065	0.018*
**Decrease in SBP (mmHg)**	20 ± 12.25	30 ± 21.85	24.57 ± 14.2	23.70 ± 9.03	20.28 ± 7.27	3.079	0.017*
**Baseline MAP (mmHg) ( just before** **induction of spinal anesthesia)**	79 ± 6.16	82 ± 2.76	83.80 ± 5.16	82.56 ± 5.41	81.69 ± 5.25	1.424	0.227
**Minimum MAP (mmHg)**	53 ± 4.16	53.17 ± 4.12	55.47 ± 4.64	57.23 ± 4.23	63.57 ± 4.11	43.119	< 0.001*
**Decrease in MAP (mmHg)**	26 ± 6.06	28.83 ± 5.74	28.1 ± 6.83	24.84 ± 6.28	18.2 ± 5.32	27.068	< 0.001*
**Duration of the longest** **hypotension episode (min)**	4.25 ± 1.71	4.33 ± 2.42	4.40 ± 2.31	3.46 ± 1.07	2.97 ± 2.45	3.294	0.012*
**Dose of vasopressor (ephedrine) (mg)**	12.25 ± 2.5	12.33 ± 5.65	12.17 ± 3.82	12.02 ± 2.84	10.91 ± 3.02	1.903	0.111
**Base deficit (mmol/l)**	18.28 ± 3.47	14.68 ± 3.28	14.42 ± 3.26	8.59 ± 2.61	6.11 ± 3.07	65.659	< 0.001*
**Fetal birth weight (gm)**	3325 ± 263	3183.3 ± 222.9	3366.7 ± 349.7	3334.2 ± 172.7	3336.6 ± 169.6	0.988	0.415
**Apgar 1 min**	7.75 ± 0.5	7.83 ± 0.98	7.70 ± 0.65	8.18 ± 0.83	8.33 ± 0.79	4.428	0.002*
**Apgar 5 min**	8.25 ± 0.5	8.17 ± 0.75	8.17 ± 0.75	8.46 ± 1.21	8.67 ± 1.27	1.376	0.243

**Fig. 1:**
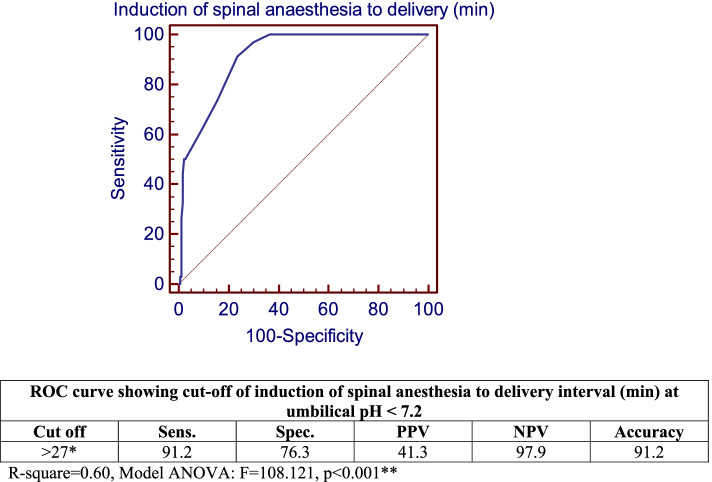
ROC Curve Showing Cut-off for Induction of Spinal Anesthesia to Delivery Interval (min) at Umbilical pH < 7.2. R-square = 0.60, Model ANOVA: F = 108.121, *p* < 0.001**

**Fig. 2:**
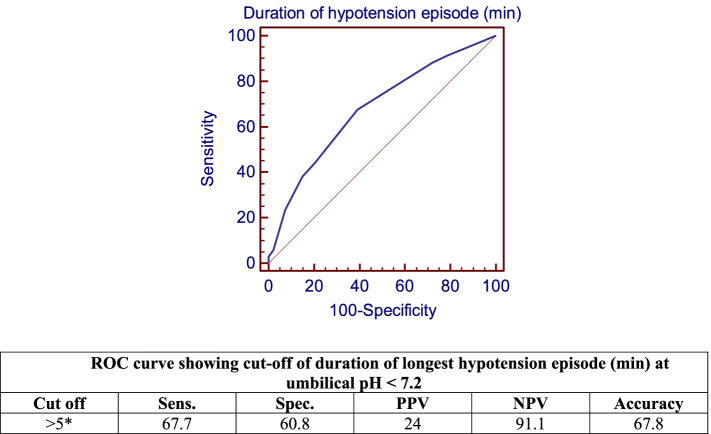
ROC Curve Showing Cut-off for Duration of Longest Hypotension Episode (min) at Umbilical pH < 7.2

**Fig. 3:**
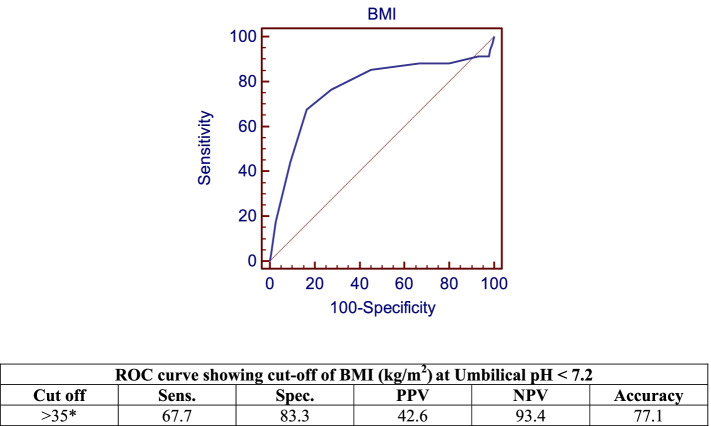
ROC Curve Showing Cut-off of BMI at Umbilical pH < 7.2

Inverse correlations between various studied intervals, BMI and longest hypotensive episode were found with umbilical pH. The greater the induction of spinal anesthesia to delivery and to skin incision intervals, the lower the umbilical pH (*r* = -0.817 and -0.706, respectively). The greater the skin incision to uterine incision interval and uterine incision to delivery interval, the lower the umbilical pH (*r* = -0.634 and -0.454, respectively). The higher the BMI and duration of the longest hypotension episode the lower the umbilical pH (*r* = -0.395 and -0.268, respectively), showing weaker correlations. All correlations showed a highly statistically significant difference (Table 5). Cut off value for induction of spinal anesthesia to delivery interval greater than 27 min predicted an umbilical pH of < 7.2 (Fig. 1). Cut off value for the duration of the longest hypotension episode greater than 5 min predicted an umbilical pH of < 7.2 (Fig. 2). Cut off value for BMI greater than 35 kg/m^2^ predicted an umbilical pH of < 7.2 (Fig. 3).

**Table 5:** Correlation between Umbilical pH with Different Studied Intervals, BMI and Duration of Longest Hypotension Episode

	**Umbilical pH**	
	***r***	***P***-value
**Induction of spinal anesthesia to delivery (min)**	-0.817	< 0.001**
**Induction of spinal anesthesia to skin incision time (min)**	-0.706	< 0.001**
**Skin incision to uterine incision time (min)**	-0.634	< 0.001**
**Uterine incision to delivery time (min)**	-0.454	< 0.001**
**BMI (kg/m2)**	-0.395	< 0.001**
**Duration of the longest hypotension episode (min)**	-0.268	< 0.001**

## Discussion

Spinal anesthesia is one of commonest and safest methods of anesthesia for cesarean delivery, yet its effect on the neonate should be further elaborated. The objective of this study was to evaluate the effect of the interval between administration of spinal anesthesia to delivery of the neonate by elective cesarean delivery on neonatal outcome, primarily neonatal acidosis. In our study, 44 (20%) neonates were born with an umbilical cord arterial pH of < 7.21. Eight (3.6%) neonates required NICU admission and one neonate required mechanical ventilation for respiratory distress. Comparing induction of spinal anesthesia to delivery interval and other studied intervals between different categories of umbilical pH showed a highly statistically significant difference (*p* < 0.001), with an interval of 40.5 ± 4.4 min at an umbilical pH of < 7 compared to an interval of 22.5 ± 3.4 min for an umbilical pH of > 7.3. Moreover, comparisons of the lowest SBP and MAP reached during the operation, the degree of drop of SBP and MAP, the duration of longest hypotension episode, the degree of base deficit and Apgar scores at 1 min between various levels of umbilical pH, all showed statistically significant differences. Patients whose neonates had an umbilical PH of < 7 had the least mean arterial pressure right before the induction of spinal anesthesia. Moreover, they had the longest induction of spinal anesthesia to delivery interval (40.5 ± 4.53 min), which was substantial, compared to patients in subsequent categories of umbilical PH (Table 3). This could explain why these patients had the lowest umbilical PH in spite of the fact that their BMI was not the highest and their minimum SBP reached was not the lowest. Furthermore, there were inverse correlations between umbilical pH and different study outcomes. Lower umbilical pH was associated with greater induction of spinal anesthesia to delivery interval, with prolonged duration of longest hypotension episode and with higher BMI. A cut-off value of more than 27 min for induction of spinal anesthesia to delivery interval, more than 5 min for longest hypotension episode and more than 35 kg/m^2^ for BMI predicted an umbilical pH of < 7.2. As shown in Table 4, patients in all categories of umbilical PH values had high BMI, yet their neonates had different values for umbilical PH ranging from too low to normal. Similarly, all patients in different categories of umbilical PH had little differences between the durations of longest hypotensive episodes. Therefore the correlations were weak compared to correlations with induction of spinal anesthesia to delivery interval. There were noticeable differences in the latter interval seen between patients in different categories of umbilical PH as shown Table 3 and therefore induction of spinal anesthesia to delivery interval showed the strongest correlation.

Rimsza et al. [3] performed a retrospective study of 527 singleton term pregnancies undergoing elective cesarean sections under spinal anesthesia. They reported that umbilical arterial pH decreased when all pre-delivery time intervals increased. A ROC curve showed that interval between beginning of spinal anesthesia to delivery more than 27 min was associated with an umbilical pH < 7.1, and an interval more than 30 min was associated with an umbilical pH < 7.0. They also showed that umbilical pH decreased when intervals from spinal start to delivery and from uterine incision to delivery increased. Other factors affecting umbilical pH were BMI, non-cephalic presentation and greatest drop of blood pressure. They also reported two cases of hypoxemic-ischemic encephalopathy.

Another retrospective analysis by Nixon et al. [7] studied the relationship of hypotension due to spinal anesthesia and intervals from anesthesia to incision and from incision to delivery with neonatal acidosis. The findings of their study revealed that both intervals were strongly related with neonatal acidosis when they were prolonged. The odds ratios for neonatal acidosis, at the 90th percentile cutoff, were 3.82 (95 percent CI, 2.03–7.19) and 2.94 (95 percent CI, 1.70–5.10), respectively. The above mentioned intervals, the use of vasopressors, and extended spinal hypotension were all linked to neonatal acidosis in multivariate analysis.

Another study [8] investigated the factors predicting umbilical pH and standard base excess in 337 elective cesarean sections performed under spinal anesthesia. They reported that interval between uterine incision to delivery, greatest drop of SBP and the effect of ephedrine use in relation to the duration of hypotension during surgery affected umbilical pH. They also recommended the use of alpha 1 agonists such as phenylephrine instead of ephedrine, which is both alpha and beta adrenergic receptor agonists, before delivery of the fetus. They stated that ephedrine crosses the placenta because it is lipid soluble, hence affecting fetal metabolism due to its beta adrenergic effect, leading to exaggerated neonatal acidosis. A meta-analysis conducted by Chao Xu et al. [9] mentioned, theoretically, that phenylephrine causes reflex bradycardia due to its vasoconstrictive effect exerted by its alpha 1 agonistic action. Reflex bradycardia could aggravate hypotension. Ephedrine, on the other hand, causes an elevation of heart rate and increases heart contractility due to its beta adrenergic action, therefore leading to an improvement of blood pressure. According to the latter meta-analysis, phenylephrine administration during elective cesarean sections was associated with lower incidence of neonatal acidosis and higher umbilical PH compared to ephedrine use.

Edwards et al. [5] conducted a retrospective multicenter study, including 5,742 women who delivered a live, non-anomalous singleton term neonate by cesarean section under spinal anesthesia. After adjustment for confounding factors, it was found that increasing BMI was associated with decreased mean pH from 7.25 to 7.22 (*P* < 0.001); proportion of neonates with pH less than 7.1 increased from 3.5% to 7.7% (*P* = 0.011); mean base deficit rose from 4.01 mmol/L to 4.83 mmol/L (*P* = 0.030), and percentage of neonates with base deficit of 12 mmol/L or more increased from 0.6% to 4.7% (*P* = 0.003). When BMI was analyzed after adjustment for confounding variables, umbilical pH declined by 0.01 (*P* < 0.001) and base deficit increased by 0.26 mmol/L (*P* = 0.005) for every 10-unit rise in BMI.

Olang et al. [10] conducted a study to determine the incidence of neonatal acidosis after cesarean section performed under spinal anesthesia, as well as the prevalence of maternal hypotension during the procedure and its relationship to neonatal acidosis. 43 babies (27.2%) were born with neonatal acidosis, (umbilical pH ≤ 7.2). However, comparison between acidotic and non-acidotic newborn babies regarding Apgar scores showed no significant difference. 28 patients (17.7%) experienced maternal hypotension defined as SBP less than 100 mmHg. Hypotension episodes did not last more than 2 min as they were readily treated by intravenous fluids and vasopressors. A duration of 1.43 min of maternal hypotension had an insignificant effect on 5-min Apgar scores of newborns. They concluded that neonatal acidosis occurring after cesarean delivery performed under spinal anesthesia had insignificant short term effects on neonates. Their results could be attributed to much frequent measuring of blood pressure at an interval of 2.5 min which allowed them to detect maternal hypotension earlier. They also used ephedrine or epinephrine to control hypotensive episodes. Also, their patients had lower BMI compared to our study.

Kana et al [11]. in which they investigated retrospectively the effect of the duration and degree of maternal hypotension on umbilical pH in 423 women undergoing cesarean section under spinal anesthesia. Mean arterial pressure less than 70 mmHg for ≥ 10 min was not a risk factor for neonatal acidosis (umbilical pH 7.304 ± 0.050 at < 10 min vs. 7.297 ± 0.045 at ≥ 10 min, *P* = 0.163). They concluded that prolonged maternal hypotension did not affect the neonatal umbilical artery pH at cesarean section under spinal anesthesia.

One strength of our study is its prospective nature. In addition, we assessed both neonatal acidosis and perinatal complications and could demonstrate that neonatal acidosis is not only related to maternal hypotension but also to the duration of hypotension during surgery. More importantly, a few studies discussed the association between various pre-delivery intervals and neonatal umbilical PH which was found to have stronger correlation than duration of hypotensive episodes and BMI. Also, induction of spinal anesthesia to delivery interval was calculated from the end of spinal block and not from the announcement of the anesthesiologist that the patient was ready. Finally, we used a lower dose of bupivicaine than most studies and recorded our results.

There are some limitations to our study. Blood pressure data was recorded at intervals of 3 to 5 min, which is less frequent than suggested in the recently published consensus statement [2]. More frequent blood pressure measurement may detect hypotension earlier, thus triggering treatment, although the impact of more frequent assessments on neonatal outcome has not been investigated. According to our results, the duration of longest hypotensive episodes had little differences between the different categories of umbilical PH, which could be attributed to intermittent blood pressure measurement and, hence, showed a weaker correlation with umbilical PH.

## Conclusion

Prolonged interval between induction of spinal anesthesia to delivery could be associated with neonatal acidosis. This could be aggravated by maternal obesity and prolonged duration of hypotension episodes during cesarean delivery.

## Data Availability

All data generated or analysed during this study are included in this published article.

## Abbreviations

SBP: Systolic blood pressure
DBP: Diastolic blood pressure
MAP: Mean arterial blood pressure
NICU: Neonatal intensive care unit
BMI: Body Mass Index
BP: Blood pressure
ANOVA: Analysis of variance
ROC: Receiver Operating Characteristic
